# Scalar Implicature is Sensitive to Contextual Alternatives

**DOI:** 10.1111/cogs.13238

**Published:** 2023-02-05

**Authors:** Zheng Zhang, Leon Bergen, Alexander Paunov, Rachel Ryskin, Edward Gibson

**Affiliations:** ^1^ Graduate School of Education, Harvard University; ^2^ Department of Brain and Cognitive Sciences Massachusetts Institute of Technology; ^3^ Department of Linguistics, University of California San Diego; ^4^ INSERM‐CEA Cognitive Neuroimaging Unit (UNICOG), NeuroSpin Center; ^5^ Department of Cognitive and Information Sciences University of California Merced

**Keywords:** Language, Scalar implicature, Rational speech‐acts, Pragmatics

## Abstract

The quantifier “some” often elicits a scalar implicature during comprehension: “Some of today's letters have checks inside” is often interpreted to mean that not all of today's letters have checks inside. In previous work, Goodman and Stuhlmüller (G&S) proposed a model that predicts that this implicature should depend on the speaker's knowledgeability: If the speaker has only examined some of the available letters (e.g., two of three letters), people are less likely to infer that “some” implies “not all” than if the speaker has examined all of the available letters. G&S also provided behavioral evidence in support of their model. In this paper, we first show that a simple extension of G&S's model (1) predicts G&S's knowledgeability effects, and in addition, (2) predicts that the knowledgeability effect will be reduced when the speaker's usage indicates numeral alternatives are available. We tested the new model's predictions in four preregistered experiments. All experiments supported the first model prediction, replicating G&S's finding of a main effect of the speaker's knowledge. Further, Experiments 2 and 4 supported the second model prediction showing that the words that a speaker tends to use affect the strength of scalar implicature that comprehenders make. In particular, when the speaker has partial knowledge (e.g., has only examined two of three letters), comprehenders think that “some” is more likely to mean “not all” when the speaker also tends to produce number words in similar sentences (e.g., “2 of today's rooms have working smoke detectors.”). These results have important ramifications for theories of meaning: the context beyond the sentence (e.g., the speaker's tendency to use particular words) affects the set of alternatives that comprehenders consider when inferring meaning.

## 1 Introduction

A fascinating feature of human language is that the interpretation of utterances varies between contexts. For example, as noted by Grice (1975), the sentence “Mr. X's … attendance at tutorials has been regular” will be interpreted in a neutral context as a straightforward claim about Mr. X's good attendance record. However, if this is the only text in a recommendation letter, it will typically receive a different (negative) interpretation. Several contextual factors can affect the interpretation of a sentence, such as the question under discussion (Groenendijk & Stokhof, 1984; Roberts, 1996); the comparison class (Kamp & Partee, 1995; Kennedy & McNally, 2005); the speaker's previous usage (Schuster & Degen, 2020); and speaker knowledgeability (Bergen & Grodner, 2012; Goodman & Stuhlmüller, 2013; Hochstein, Bale, Fox, & Barner, 2016; Dieuleveut, Chemla, & Spector, 2019; Spychalska, Reimer, Schumacher, & Werning, 2021).

The current work focuses on the role of *alternative utterances* in arriving at an interpretation during the process of language understanding. In many contexts, the sentence “Some of the letters have checks inside” will trigger a scalar implicature: the inference that not all of the letters have checks inside (Gazdar, 1979; Grice, 1975; Grice, 1978; Horn, 1972). The listener will typically draw this inference because the speaker could have used an alternative word, “all” instead of “some,” if indeed all of the letters had checks inside. Alternative utterances play a similar role in all other areas of pragmatic inference, with the listener inferring the speaker's intended meaning based on what the speaker chose not to say.

A prominent line of research has proposed that the set of alternative utterances considered during pragmatic reasoning is determined by the grammatical structure of a sentence (Fox & Katzir, 2011; Horn, 1972; Katzir, 2007). Under these proposals, alternative utterances are generated through lexical substitution into the sentence that the speaker produces. These proposals describe algorithms that determine which words can be substituted into the original sentence, and which words should be replaced.

Beyond the syntax, the visuospatial context in which a sentence is interpreted also affects the set of plausible alternatives for listeners. For instance, when viewing a display in which some objects constitute a contrast set (e.g., a big cup and a small cup) and others are unique (e.g., a single big spoon), listeners anticipate that a sentence containing a scalar adjective, “Hand me the big…,” will refer to the item in a contrast set (the big cup) rather than the singleton item, likely reasoning that the speaker would not have used “big” if the ultimate referent were not in a contrast set (Sedivy, Tanenhaus, Chambers, & Carlson, 1999). Similarly, when considering candidate referents of sentences, listeners take into account what the speaker can and cannot see (e.g., Hanna, Tanenhaus, & Trueswell, 2003; Heller, Grodner, & Tanenhaus, 2008) as well as the informational goals of the speaker (e.g., questions vs. statements; Brown‐Schmidt, Gunlogson, & Tanenhaus, 2008).

In addition, social information about the speaker in the larger conversational context can impact how alternatives are weighed. For instance, when listening to a speaker who repeatedly uses scalar modifiers infelicitously—including a scalar modifier when referring to a singleton or omitting it when it is needed for disambiguation—listeners become less likely to anticipate that the referent is one that belongs to a contrast set (Grodner & Sedivy, 2011; Ryskin, Kurumada, & Brown‐Schmidt, 2019). Plausibly, when the speaker is unreliable, any object in the visual context—whether in a contrast set or not—can be referred to with the same set of alternative utterances (e.g., a modified noun phrase or a bare noun phrase).

In order to investigate how alternatives are considered in different contexts, severa researchers have focused on the processing of the word “some” in contexts with and without numeral word alternatives. More specifically, when a speaker uses numerals in other sentences in the conversational context, this impacts the listener's processing of “some.” Grodner, Klein, Carbary, and Tanenhaus (2010) were the first to investigate this phenomenon experimentally, collecting naturalness ratings for noun phrases initiated by “some of,” both with and without number terms included in the context. They found that including number words in the context marginally reduced the naturalness of noun phrases initiated by “some of ” and hypothesized that the existence of the more natural number words might reduce the felicity of the implicature of “some of.” Degen and Tanenhaus (2015) also found reduced naturalness ratings for noun phrases initiated by “some” when the context contained numerals, and additionally, Degen and Tanenhaus (2016) showed that people were slower to interpret “some” if the speaker had previously used numerical utterances, such as “two” or “three” (see also Chierchia, Crain, Guasti, Gualmini, & Meroni, 2001, and Skordos & Papafragou, 2014, for relevant developmental evidence using different types of alternatives).

The goal of the present study is to investigate the integration of information about alternative utterances with a second important contextual factor affecting how utterances are interpreted: *knowledgeability*. In a landmark study, Goodman and Stuhlmüller (2013) (henceforth G&S) showed that experimental participants were less likely to infer a scalar implicature (“some” meaning “not all”) when the speaker had only partial knowledge (cf. Sauerland, 2004; Van Rooij & Schulz, 2004). G&S investigated the effect of speaker knowledgeability through a paradigm in which participants read scenarios like (1), in one of two conditions “full knowledge” or “partial knowledge”:

(1) Context:

Letters to Laura's company almost always have checks inside. Today Laura received 3 letters. Laura tells you on the phone:

- *Full knowledge condition*: **I have looked at 3 of the 3 letters**. Some of the letters have checks inside.
- *Partial knowledge condition*: **I have looked at 2 of the 3 letters**. Some of the letters have checks inside.

In the full knowledge condition (1a), the speaker (“Laura”) has access to all knowledge relevant to their communication goals: knowledge of all three letters that she has received. The partial knowledge condition is identical, except that the speaker only has access to part of the relevant information: two of the three letters. G&S measured the strength of the scalar implicature triggered by “some” in these conditions in the final sentence of the texts: “*Some of the letters have checks inside*” in (1) by asking experimental participants to bet portions of $100 on how many letters had checks inside. G&S found that the scalar implicature triggered by “some” was sensitive to the degree of speaker knowledgeability. When the speaker was only partially knowledgeable as in (1b), experimental participants bet more money on all three letters having checks inside, suggesting that they inferred a weaker scalar implicature. When the speaker had looked at all three letters, the participants inferred a stronger scalar implicature: they were more likely to infer that not all of the letters had checks inside, so they bet more money on there being one or two letters with checks inside (rather than all three).

G&S observed that this result is hard to account for within the grammatical approach to scalar implicature because only the context is changing among the knowledgeability conditions. G&S proposed a solution within the rational speech‐acts (RSA) framework proposed by Frank and Goodman (2012). Standard RSA assumes that speakers are fully knowledgeable about the world. As described in more detail in the next section, G&S relaxed this assumption, allowing for a speaker who is only partially knowledgeable. In this model, when the speaker did not know the contents of all the letters (e.g., they only opened two of the available three) and then they said “Some of the letters have checks inside,” the listener was less likely to make the scalar implicature that not all of the letters had checks inside.

A particularly interesting case arises when we consider the effects of both knowledgeability and whether the speaker uses alternative words for “some,” such as number words, in the surrounding context. A natural extension of the model proposed by G&S predicts that having alternative words available *would increase the scalar implicature in the low‐knowledge condition*. That is, if the speaker has only opened two of the three letters, and also often uses words like “two” or “three,” then the listener should infer that “some” could mean “not all” more than in the baseline low‐knowledge context. According to the grammatical approach, the speaker's tendency to use numerals should not impact the scalar implicature. The flexible integration of these distinct contextual cues is readily accommodated within the RSA framework and is the focus of the current study.

We first propose a model in the RSA framework (e.g., Goodman & Frank, 2016), extending the model from G&S to allow for variation in the alternative utterances which speakers and listeners reason about. This model predicts (1) that the scalar implicature associated with “some” will be (partly) canceled due to the speaker's incomplete knowledge (G&S), and more importantly (2) that this knowledgeability effect will be reduced when the speaker's usage indicates that numeral alternatives are available. In other words, the model accommodates two kinds of contextual effects on language interpretation: a social‐cognitive inference of the speaker's knowledge state (as shown in G&S), and, novelly, a contextual constraint on the set of quantifiers available to the speaker. These predictions are evaluated in a set of experiments which manipulate the speaker knowledgeability and the set of alternative utterances salient to the speaker.

## 2 An RSA model

The model we propose extends G&S's model, starting with a literal listener *L*_0_, who interprets utterances according to their literal meanings:

(2) $$L_{0}\left(w|u\right)\propto P\left(w\right)1_{w\in ⟦u⟧}$$

*L*_0_ (*w*|*u*) represents the probability that this listener assigns to world *w*, given that they have heard utterance *u*. Here, *P*(*w*) is the prior probability of world *w*, and [*u*] is the semantic interpretation of utterance *u*. (2) indicates that the literal listener filters out worlds which are literally incompatible with the perceived utterance *u* (e.g., if the utterance is “some,” a world in which 0 of the letters have checks inside is literally incompatible), and weights the remaining worlds by their prior probability.

The speaker *S*_1_ has a particular knowledge state *s*, and they want to communicate this knowledge state to the listener *L*_0_. The model assumes that *s* consists of a set of worlds, that is, the worlds consistent with what the speaker knows. The utility of an utterance *u* is given by (3):

(3) $$U_{1}\left(u|s\right)=\sum_{w\in s}P\left(w|s\right)\log L_{0}\left(w|u\right)$$

This utility function encodes the speaker's preferences over utterances. Utterances are preferred when they lead the listener *L*_0_ to assign a higher probability to the worlds in the speaker's knowledge state. The speaker's distribution over the choice of utterance is determined by a softmax decision rule (4):

(4) $$S_{1}\left(u|s,\;A\right)=\frac{e^{U_{1}\left(u|s\right)}}{\sum_{u^{\prime }\in A}e^{U_{1}\left(u^{\prime }|s\right)}}$$

The term A is the set of alternative utterances available to the speaker. The speaker's decision rule implies that they are more likely to choose utterances which receive higher utility.

The pragmatic listener, *L*_1_, uses their model of the speaker *S*_1_ to interpret utterances. Given an utterance *u*, they consider which knowledge states would have made the speaker most likely to choose that utterance. This reasoning is formalized using Bayes’ rule (5):

(5) $$L_{1}\left(w|u\right)\propto \sum_{A}P\left(A\right)\sum_{s}P\left(s|w\right)S_{1}\left(u|s,\;A\right)$$

**Fig. 1:**
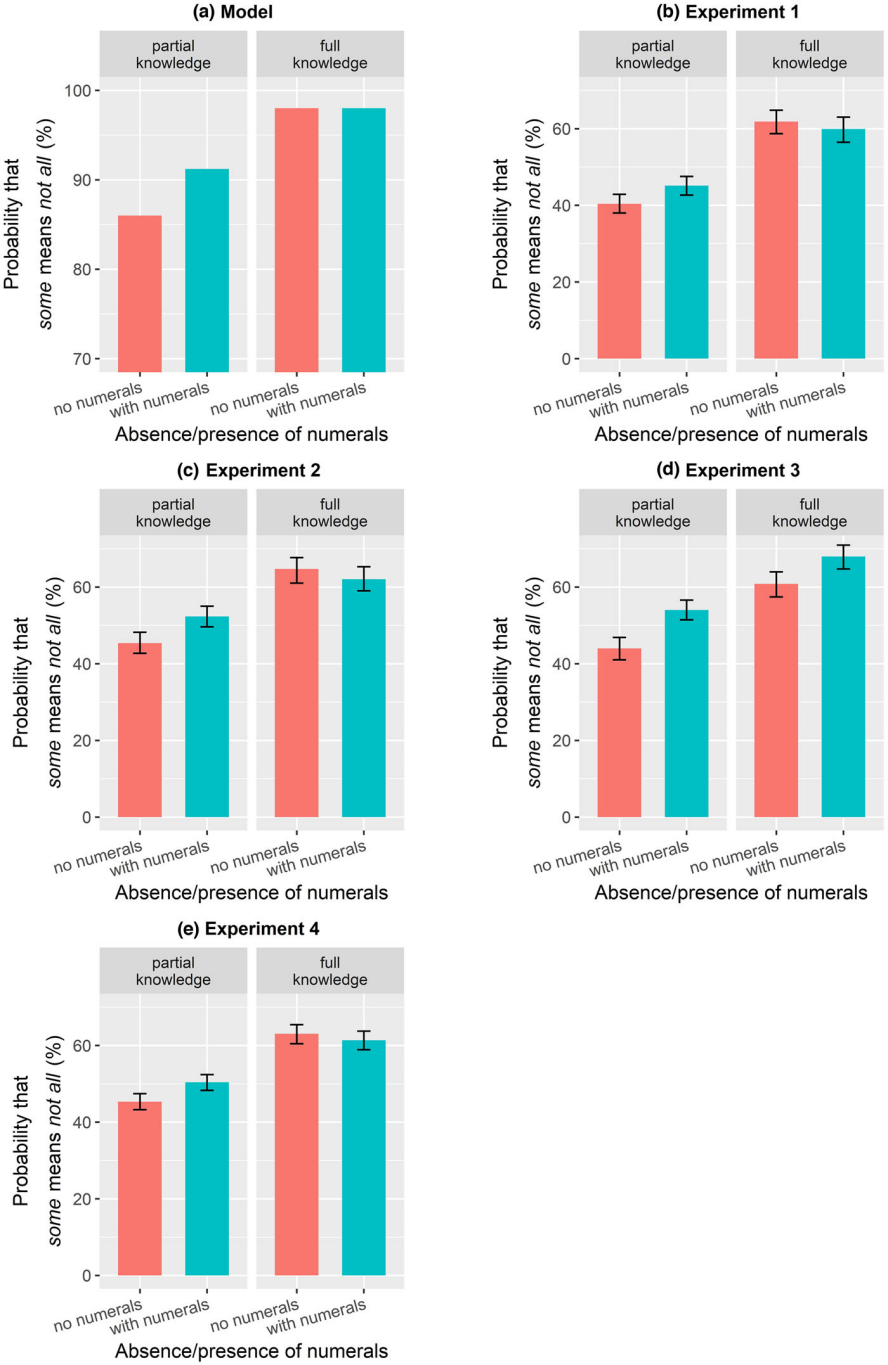
(a) Model predictions for the four experimental conditions. As in Goodman and Stuhlmüller (2013), the model predicts a main effect of knowledgeability, such that the scalar implicature (high probability that *some* means *not all*) is stronger for the full knowledge condition than for the partial knowledge condition. More importantly, the model predicts an interaction between knowledgeability and alternative availability: The scalar implicature is predicted to be equally strong in the two full knowledgeability conditions, but there should be a difference in the partial knowledge conditions, such that it should be stronger where numeral alternatives are available. (b–e): Results of Experiments 1–4. Mean participants’ ratings of the probability of *not all* items having the property, varying the speaker knowledgeability and the existence of contextual numerals. Note that higher probability numbers reflect more endorsement of the “some but not all” interpretation of “some.” Error bars represent 95% confidence intervals. Note that the scale for the model differs from that for the experiments, because the model parameters were not optimized to fit experimental results. Critically, the interaction is robust across a range of model parameters. In particular, we present the results while assuming a uniform prior distribution (perhaps the simplest assumption), whereas, in the experiments, it was stated that most elements in the set have the target property. Thus, if we had assigned more weight to the “all” world (as in the example contexts in our experiment), we would have got a closer fit to the experimental data.

Here, *L*_1_ (*w*|*u*) is the probability that *L*_1_ assigns to world *w* given utterance *u*, and *P*(*s*|*w*) is the probability that the speaker has knowledge state *s* given that the true world is *w*. *P(A)* is the probability that the listener assigns to the set of alternatives *A*. When the listener is certain that the set of alternatives contains numerals, P(A = A_no numerals_) = 0 and P(A = A_with numerals_) = 1. When the listener is certain that the set of alternatives does not contain numerals, P(A = A_no numerals_) = 1 and P(A = A_with numerals_) = 0. Values of *P(A)* between 0 and 1 reflect a listener who is uncertain about the set of alternatives being used by the speaker. This is a valuable generalization, suggested by a reviewer, if, for example, in future work, the model is explicitly fitted to behavioral data and/or if *P(A)* is manipulated between 0 and 1. Here, however, our results only speak to the extreme settings of *P(A)* to 0 and 1. Fig. 1(a) shows model predictions for these cases; for complete model predictions including intermediate cases, see Fig. A1 in the online Appendix.

Throughout, we assume that the set of worlds W = {0,1,2,3}, where these numbers represent the number of objects with some property (e.g., the number of letters containing checks). When the speaker says they have seen three of the three objects (hence complete knowledge), they know exactly which world they are in. When the speaker has only partial access (hence partial knowledge), they have only observed a subset of the objects, and therefore, their observations are consistent with multiple worlds.

In our experiments, we manipulate the set of alternative utterances *A* available to the speaker, by giving the listeners a context either with numerals or without numerals. In both conditions, our model assumes that there is a core set of alternatives available: *none*, *some*, and *all*. In the non‐numeral condition, these exhaust the set of alternatives. In the with‐numeral condition, there are three additional alternatives available: *one*, *two*, and *three*. No other positive integer number words are possibly consistent with the speaker's context, because there are only three objects in each scenario. With respect to the model, we assume a lower‐bounded semantics for these numerals (e.g., two means “at least two”), and that the exact interpretations (e.g., “exactly two”) are obtained through a scalar implicature (Horn, 1972; Gazdar, 1979; Levinson, 1983; Horn, 1989).

The model predicts an interaction between knowledgeability and the strength of the scalar implicature associated with *some*, as shown in Fig. A1 in the online Appendix. Suppose first that the speaker is fully knowledgeable. In this case, the listener *L*_1_ knows that if the speaker has observed world 3 (in which all of the letters contain checks), then they would have said *all*; the utterance *all* is the most informative in this world. When *L*_1_ hears *some* in this case, they will strongly infer that the speaker did not observe this world, and that not all of the letters contain checks. This reasoning holds for both sets of alternatives (i.e., the with‐numeral set of alternatives and the non‐numeral set of alternatives). Hence, the probability that *some* means *not all* (the scalar implicature) is high for each set of alternatives.

Next, suppose that the speaker is only partially knowledgeable, in particular, that they have observed two of the three letters. In this case, the set of alternative utterances will influence how the listener interprets *some*. Suppose first that the speaker has the numeral alternatives available. The speaker then may say *two* if both of the letters that they have observed contain checks; this is more informative than the other alternatives (*some* and *one*). When the listener *L*_1_ hears *some*, they will infer that it is less likely that both letters observed by the speaker contain checks than the baseline suggested (which is that most letters contain checks). Hence, it is unlikely that all three letters (including the one not yet observed) contain checks: “some” is quite likely to be interpreted as “not all.”

In the absence of the numeral alternatives, the scalar implicature from *some* will weaken. The speaker should choose the utterance *some* regardless of whether only one or both of the observed letters contain checks. This is the only available utterance which is literally compatible with their observations. The listener, therefore, does not gain any information about whether one or two of the observed letters contain checks, and, therefore, does not gain any information about whether all of the letters contain checks. Hence, this model predicts that in the partial knowledgeability condition, *some* is more likely to be interpreted as “some but not all” when the speaker has the numeral alternatives available.

## 3 Experiments

We present the results of four experiments. The results from the four experiments are visualized in Fig. 1, along with the predictions of the model above. We also present the results from the four experiments in table form in Table 1.

**Table 1:** Summary of the model prediction, the crucial difference in the experimental design, and the significant experimental results

	Experimental design			Four experimental conditions				Crucial prediction/results		
	number of trials			partial knowledge		full knowledge				
	target	exposure	Range of the numerals in the exposure trials	no numerals	with numerals	no numerals	with numerals	main effect of knowledgeability	main effect of contextual numerals	interaction between knowledgeability and contextual numerals
Model				86	91.2	98	98	predicted	not predicted	predicted
Exp. 1	8	16	2–22	40.4 (27.2)	45.1 (28.7)	61.9 (35.9)	59.9 (37.2)	*β* = 18.08, *SE* = 1.95, *t* = 9.26, * *p* < .001		*β* = –6.54, *SE* = 3.88, *t* = –1.69, . *p* = .094
Exp. 2	8	24	2–25	45.4 (30.7)	52.3 (30.0)	64.7 (37.1)	62.0 (35.7)	*β* = 14.60, *SE* = 1.91, *t* = 7.65, * *p* < .001		*β* = –9.76, *SE* = 3.95, *t* = –2.47,* *p* = .018
Exp. 3	8	24	1–5	44.0 (31.6)	54.0 (30.7)	60.8 (37.8)	68.0 (35.4)	*β* = 15.41, *SE* = 1.94, *t* = 7.93, * *p* < .001	*β* = 8.60, *SE* = 3.25, *t* = 2.65, * *p* =.009	
Exp. 4	8	24	2–25	45.3 (29.9)	50.4 (30.2)	63.1 (34.1)	61.3 (33.5)	*β* = 14.24, *SE* = 1.34, *t* = 10.62,* *p* < .001		*β* = –6.90, *SE* = 2.76, *t* = –2.50, * *p* = .017

*Note*. Numbers under “Four experimental conditions” represent mean (standard deviation in parentheses) values of participants’ rating of the probability of *not all* items having the property (in %). "*" indicates p<0.05 "." indicates p<0.1.

### 3.1 Experiment 1

Experiment 1 was conducted to test the predictions of the proposed RSA model. More specifically, it was intended to (1) replicate G&S's finding that the speaker's knowledge has an effect on the comprehender's interpretation of scalar implicature, and (2) test for an interaction between the speaker's knowledge and the existence of contextual numerals. All materials and analyses of Experiment 1 were preregistered and are available at https://osf.io/39nrj/.

#### 3.1.1 Method

##### 3.1.1.1 Participants

Participants were recruited on Amazon.com's Mechanical Turk and completed the experiment for $2.5 payment. We used the Turkolizer software from Gibson, Piantadosi, and Fedorenko (2011). The experimental tasks were available to participants who had (i) an IP address inside the United States, (ii) previously completed more than 100 tasks on Mechanical Turk, (iii) an approval rate above 95%, and (iv) had not participated in similar experiments before.

We kept collecting data until we obtained 240 participants who met all the requirements listed as follows. (i) Participants’ native language was English (based on self‐report). (ii) They completed more than 90% of the target questions (i.e., the probability rating questions). (iii) Their accuracy on comprehension questions in the target trials was 100%. (iv) They came from the United States (based on self‐report and IP address).

##### 3.1.1.2 Materials, design, and procedure

This experiment was based on G&S's paradigm, using materials similar to those used by G&S in (1). We first describe the method we used, and then we outline the differences with G&S's.

Participants were told that they would read 24 scenarios, and that for each scenario, they would answer Yes/No questions and give a probability rating (a number from 0% to 100%, indicating the probability of all items having a target property). The 24 scenarios consisted of 8 target trials and 16 exposure trials. The order of the 24 items was pseudo‐randomized for each participant with the constraints that the first item was an exposure trial and two target items did not directly follow each other.

Each scenario given to the participant was composed of three parts: a context; a statement about the context; and three questions that the participant was required to answer.

In target trials, the context stated that a speaker needed to investigate three objects that could have a certain property. Each scenario was followed by a statement in which the speaker declared how many of the objects they had observed—either three of three (full knowledge) or two of three (partial knowledge)—and stated that “some” objects had the property as in (6) (based closely on (1) above):

(6) Example target trial:

*Context*:

Letters to Laura's company almost always have checks inside. Today Laura needs to find out whether 3 of the letters have checks inside. Laura tells you on the phone:

*Statement*:

(a) Full knowledge condition:

I have now looked at 3 of the 3 letters, and given what I saw, I can tell you that some of today's letters have checks inside.

(b) Partial knowledge condition:

I have now looked at 2 of the 3 letters, and given what I saw, I can tell you that some of today's letters have checks inside.

Each participant received 4 partial knowledge target trials and 4 full knowledge target trials.

In exposure trials, the context stated that the speaker needed to investigate *n* objects (3 ≤ *n* ≤ 22) that could have a certain property, as in (7) and (8). There were two types of exposure trials: “with numerals” exposure trials and “no numerals” exposure trials.

In the “with numerals” exposure trials, the speaker used numerals. They first used numerals to declare how many of the objects they had observed—either “x of x [objects]” (full knowledgeability) or “y of x [objects],” where y<x (partial knowledgeability)—, and then made a statement using numerals (e.g., “I can tell you that 3 of today's rooms have working smoke detectors.”). Whereas, in the “no numerals” exposure trials, the speaker did not use numerals. They first indicated how many of the objects they had observed without using numerals—either “the [objects]” (indicating full knowledgeability), or “many/several of the [objects]” (indicating partial knowledgeability)—and then made a general statement without numerals (e.g., “I can tell you that the new interns are really irresponsible.”). Contrary to the target trials, the speaker in the exposure trials did not say “some.”

Half of the exposure trials had a speaker with full knowledgeability; the other half had a speaker with partial knowledgeability. The numeral type of exposure trials (i.e., “with numerals” or “no numerals”) was manipulated between subjects. Participants were randomly assigned to one of the two exposure conditions.

(7) *Example exposure trial (with full knowledgeability)*:

*Context*:

The rooms in Mary's office building almost always have working smoke detectors. Today Mary is supposed to check the smoke detectors in 3 rooms. Mary tells you on the phone:

*Statement*:

(a) No numerals version

I have now looked at the rooms, and given what I saw, I can tell you that I am very satisfied with the result.

(b) With numerals version

I have now looked at 3 of the 3 rooms, and given what I saw, I can tell you that 3 of today's rooms have working smoke detectors.

(8) *Example exposure trial (with partial knowledgeability)*:

*Context*:

The medical records in Jonah's hospital are almost always correctly labeled. Today Jonah needs to check whether 15 medical records are correctly labeled. Jonah tells you on the phone:

*Statement*:

(a) *No numerals version*

I have now checked many of the medical records, and given what I saw, I can tell you that the new interns are really irresponsible; they messed things up.

(b) *With numerals version*

I have now checked 13 of the 15 medical records, and given what I saw, I can tell you that 11 of today's records are correctly labeled.

In the question phase (as in (9)), the first two questions were designed to confirm that the participant had read the materials carefully and fully understood what was going on in the given scenario. Thus, the first two questions had correct answers. The third question asked the participant the probability of *all* the items having the property.

(9) *Example Questions (corresponding to (7))*:

(a) Did Mary look at all of today's rooms? *(correct answer: yes)*
(b) Did Mary say that none of today's rooms have working smoke detectors? *(correct answer: no)*
(c) Assuming that the speaker is providing an accurate description of her knowledge, how likely is it that all of today's rooms have working smoke detectors? Please provide a probability between 0 and 100 percent: ____%

There are several differences between our experiment and G&S's original experiment. First, G&S had only six items, whereas we have 24. Second, G&S had three levels of knowledgeability (having observed 1, 2, or 3 of the items), whereas we reduced the levels of knowledgeability to two (partial and full knowledge) because the critical comparison only requires full *versus* partial knowledge conditions. This simplification also enables a balanced design, in which the full and partial knowledge conditions occur with equal frequency. Third, G&S's materials included low‐frequency, unfamiliar objects and properties, such as in “Corendula seeds almost always sprout within a day when put into water.” In the replication, we only used familiar objects and situations, which were plausible with the “almost always” context set up in the first sentence. Fourth, G&S used two questions in each trial to test the prior belief, while we directly instructed the participants to assume that the speaker was providing an accurate description of their knowledge. Fifth, G&S used a betting measure in their experiment, asking participants to divide $100 across four scenarios. In contrast, we asked participants to provide a single probability estimate in percentage of all the items having the property. Consequently, participants only had to provide one probability measure for each trial, not four, as in G&S's method.

Critically, we included exposure trials in order to manipulate the base rate frequency of numerals in the experimental context across the two numeral conditions. The goal of this manipulation was to test, across conditions, whether the probability of drawing the scalar implicature is jointly influenced by an interaction of two pragmatic factors: the speakers’ inferred knowledgeability and the background probability that speakers might use numerals to describe their observations.

#### 3.1.2 Results

We only analyzed participants’ answers in the target trials. Participants’ answers in the exposure trials were excluded from the data analysis.

To obtain a more straightforward interpretation of the results as the strength of endorsement of the “some but not all” implicature of “some,” we transformed participants’ responses by subtracting their probability rating value from 100% in data analyses. For example, a response of 80% (i.e., the participant thought the probability of all of today's letters having checks inside was 80%) was transformed to 20% (i.e., they thought the probability of not all of today's letters having checks inside was 20%). After the transformation, higher probability numbers reflect a greater endorsement of the “some but not all” implicature of “some.” Hence, the transformed participants’ response in the target trials was the dependent variable we analyzed.

Fig. 1(b) shows the mean rating of the probability of not all items having the property across knowledgeability conditions and in both exposure conditions.

Effects of speaker knowledgeability and exposure to numeral alternatives on probability ratings were assessed via linear mixed‐effects regression using the lme4 package in R (Bates, Mächler, Bolker, & Walker, 2015). Knowledgeability (partial = –0.5, full = 0.5) and exposure (no numerals = –0.5, with numerals = 0.5), along with their interaction, were entered as contrast‐coded fixed effects. Varying intercepts were included for participants and items with varying slopes for knowledgeability by participants and varying slopes for all effects of interest (the two main effects and their interaction) by items.^1^

Table 1 shows the mean participants’ rating by four experimental conditions and the standard deviation. Table 2 shows the regression table of the fixed effects (see Table A1 in the online Appendix for random effects).

**Table 2:** The regression table of the fixed effects of Experiment 1

	Estimate	Std. error	df	*t*‐value	*p*‐value
Intercept	51.827	1.681	83.190	30.822	<2e‐16
Knowledgeability	18.076	1.952	168.096	9.260	<2e‐16
Exposure (numerals)	1.401	3.213	133.763	0.436	.6635
Knowledgeability × Exposure	–6.544	3.881	177.806	–1.686	.0935

The probability ratings were lower in the partial knowledge condition than in the full knowledge condition (*β* = 18.08, *SE* = 1.95, *t* = 9.26, *p* < .001), which suggested that participants who knew the speaker had limited knowledge (the “partial knowledgeability” condition, *M* = 42.77, *SD* = 28.08) thought there was a smaller likelihood of not all the items having the property than those who knew the speaker had full knowledge (the “full knowledgeability” condition, *M* = 60.89, *SD* = 36.55).

There was no main effect of the contextual numerals on the probability ratings (*β* = 1.40, *SE* = 3.21, *t* = 0.44, *p* = .66).

The analyses also indicated that the interaction between the speaker knowledgeability and the availability of the contextual numerals was not significant (*β* = –6.54, *SE* = 3.88, *t* = –1.69, *p* = .0935).^2^

#### 3.1.3 Discussion

The probability ratings were lower in the partial knowledge condition than in the full knowledge condition. This replicated the results of G&S and was predicted by our RSA model: The speaker knowledgeability has an effect on the comprehender's derivation of scalar implicature, such that when the speaker has incomplete knowledge, the “some but not all” implicature of “some” is (partly) canceled.

Numerically, the difference between full and partial knowledgeability was reduced when participants were exposed to numerals, but the predicted interaction between knowledgeability and exposure was not significant. There are several possible issues with the experiment's methodology. First, the hypothesized interaction may only occur if numeral alternatives are salient to participants. The methodology in Experiment 1 may not have achieved this, as participants may not have observed the numeral alternatives frequently enough in the exposure trials. Second, in pilot work by Paunov and Gibson, it was discovered that the knowledgeability effect was not present when there were no filler/exposure trials, suggesting that participants do not read the materials naturally when too many similar target materials are present. We, therefore, increased the number of exposure trials in Experiment 2.

### 3.2 Experiment 2

In Experiment 2, we increased the ratio of exposure trials to target trials from 2:1 to 3:1, keeping the range of numerals similar to Experiment 1 (2–22 and 2–25, respectively). We expected the change would make the No Numerals versus With Numerals manipulation more salient and the effect size of the hypothesized interaction larger. All materials and analyses for Experiment 2 were preregistered and are available at https://osf.io/39nrj/.

#### 3.2.1 Method

*Participants* were recruited on Amazon.com's Mechanical Turk and completed the experiment for $3 payment. We kept collecting data until we had 240 participants who met the inclusion criteria. Data inclusion criteria were the same as those of Experiment 1.

*Design, materials, and procedure* were all similar to Experiment 1, except that eight more exposure trials were added in each contextual numerals condition. Hence, each participant received 32 trials (8 target trials + 24 exposure trials).

#### 3.2.2 Results

Fig. 1(c) shows the mean participants’ rating of the probability of *not all* items having the property across knowledgeability conditions and in both exposure conditions.

Effects of speaker knowledgeability and exposure to numerals on probability ratings were assessed via linear mixed‐effects regression using the lme4 package in R (Bates et al., 2015). Knowledgeability (partial = –0.5, full = 0.5) and exposure (no numerals = –0.5, numerals = 0.5), along with their interaction, were entered as contrast‐coded fixed effects. Varying intercepts were included for participants and items with varying slopes for knowledgeability by participants and varying slopes for all effects of interest (the two main effects and their interaction) by items.^3^ Table 3 shows the regression table of the fixed effects (see Table A1 in the online Appendix for random effects).

**Table 3:** The regression table of the fixed effects of Experiment 2

	Estimate	Std. error	df	*t*‐value	*p*‐value
Intercept	56.148	1.692	122.823	33.178	<2e‐16
Knowledgeability	14.595	1.908	47.821	7.651	7.61e‐10
Exposure (numerals)	2.154	3.351	101.739	0.643	.5218
Knowledgeability × Exposure	–9.764	3.950	38.729	–2.472	.0179

The probability ratings were lower in the partial knowledge condition (*M* = 48.85, *SD* = 30.51) than in the full knowledge condition (*M* = 63.39, *SD* = 36.41; *β* = 14.60, *SE* = 1.91, *t* = 7.65, *p* < .001). There was no main effect of the exposure numerals on the probability ratings (*β* = 2.15, *SE* = 3.35, *t* = 0.64, *p* = .52). Crucially, the interaction between speaker knowledgeability and contextual numerals was significant (*β* = –9.76, *SE* = 3.95, *t* = –2.47, *p* = .018). There was a larger effect of knowledgeability on scalar implicature in the context without numerals than in the context with numerals.^4^

We also conducted follow‐up tests of the effect of contextual numerals within each knowledgeability condition, by dummy‐coding the knowledgeability conditions. When the speaker had only partial knowledge, the probability ratings were higher in the context with numeral alternatives than in the context without numeral alternatives (*β* = 7.04, *SE* = 3.32, *t* = 2.12, *p* = .036).^5^ When the speaker had full knowledge, the probability ratings in the context with numeral alternatives were not significantly different from those in the context without numeral alternatives (*β* = –2.71, *SE* = 4.22, *t* = –0.64, *p* = .52).^6^

#### 3.2.3 Discussion

The probability ratings were lower in the partial knowledge condition than in the full knowledge condition, thus replicating Experiment 1 and G&S, and as predicted by our RSA model.

This experiment also provided evidence for the predicted interaction between knowledgeability and alternative availability. The follow‐up tests furthermore showed that the strength of the scalar implicature did not differ in the full knowledgeability conditions, but was stronger in the partial knowledge case when numeral alternatives were available. These results were all in line with the predictions of our RSA model.

### 3.3 Experiment 3

In Experiments 1 and 2, the numerals introduced during exposure ranged from 3 to 25. This variety was included to make the purpose of the study less evident and avoid strategic behavior (e.g., always responding with the same value for target trials, with “some”). In Experiment 3, we altered the exposure trials to only include numerals in the range from one to five, to test for the generalizability of the result across different exposure numerals. This was done in order to evaluate whether increasing the frequency of the numerals one through five would increase the salience of these alternative utterances for participants. Given the increased salience of these alternatives, the experimental manipulation could be strengthened, and a larger interaction between exposure type and knowledgeability might be expected. All materials and analyses for Experiment 3 were preregistered and are available at https://osf.io/39nrj/? .

#### 3.3.1 Method

*3.3.1.1 Participants* were recruited on Amazon.com's Mechanical Turk and completed the experiment for $3 payment. We kept collecting data until we obtained 240 participants who met the preregistered requirements. Participant's qualification requirements and data inclusion criteria were the same as those of Experiment 2.

*3.3.1.2 Design, materials, and procedure* were all similar to Experiment 2, except that large numbers in the exposure trials were replaced by small numbers (i.e., 1, 2, 3, 4, or 5).

#### 3.3.2 Results

Fig. 1(d) shows the mean rating of the probability of *not all* items having the property across knowledgeability conditions and in both exposure conditions.

Effects of speaker knowledgeability and exposure to numerals on probability ratings were assessed via linear mixed‐effects regression using the lme4 package in R (Bates et al., 2015). Knowledgeability (partial = –0.5, full = 0.5) and exposure (no numerals = –0.5, with numerals = 0.5), along with their interaction, were entered as contrast‐coded fixed effects. Varying intercepts were included for participants and items with varying slopes for knowledgeability by participants and varying slopes for all effects of interest (the two main effects and their interaction) by items.^7^ Table 4 shows the regression table of the fixed effects (see Table A1 in the online Appendix for random effects).

**Table 4:** The regression table of the fixed effects of Experiment 3

	Estimate	Std. error	df	*t*‐value	*p*‐value
Intercept	56.709	1.902	55.066	29.815	<2e‐16
Knowledgeability	15.412	1.943	60.167	7.932	6.29e‐11
Exposure (numerals)	8.602	3.251	236.285	2.646	.0087
Knowledgeability × Exposure	–2.895	3.880	98.472	–0.746	.4573

As in Experiments 1 and 2, the probability ratings were lower in the partial knowledge condition (*M* = 49.01, *SD* = 31.55) than in the full knowledge condition (*M* = 64.41, *SD* = 36.76; *β* = 15.41, *SE* = 1.94, *t* = 7.93, *p* < .001).

Contrary to the model predictions, there was a main effect of the contextual numerals on the probability ratings (*β* = 8.60, *SE* = 3.25, *t* = 2.65, *p* = .0087), which suggested that participants who were exposed to numeral alternatives (the “with numerals” condition, *M* = 61.01, *SD* = 33.85) thought there was a larger likelihood of not all the items having the property than those who were not exposed to numeral alternatives (the “no numerals” condition, *M* = 52.41, *SD* = 35.81).

The interaction between speaker knowledgeability and contextual numerals was not significant (*β* = –2.90, *SE* = 3.88, *t* = –0.75, *p* = .46).^8^

#### 3.3.3 Discussion

As in Experiments 1 and 2, the probability ratings were lower in the partial knowledge condition than in the full knowledge condition, thus replicating G&S, as predicted by our RSA model.

Unexpectedly, we found a main effect of the contextual numerals on the participants’ interpretation of “some,” which was not predicted by our RSA model. Furthermore, we did not find the predicted interaction.

Thus, it appears that the predicted interaction is not robust to any sets of numeral exposure trials. It may be that having too many similar materials—always talking about small numbers between 1 and 5—induces some kind of unnatural strategy in our task or attentional effect, such that the participants do not (subconsciously) notice that the target trials are different in the experimentally relevant ways. Although this might be interesting to explore in future work, we put these effects aside, and instead tried to see how robust our original findings in Experiment 2 were, where the exposure trials were more varied.

### 3.4 Experiment 4

In Experiment 3, the exposure trials were more similar to the target trials than in Experiments 1 and 2. We speculate that this similarity may have induced some kind of strategic behavior. Experiment 4 was designed as a direct replication of Experiment 2, where the exposure trials were varied, and which showed the predicted interaction between speaker knowledgeability and contextual numerals. We first conducted a power analysis of Experiment 2, so that we knew how many participants were needed to replicate the effects. All materials and analyses for Experiment 4 were preregistered and are available at https://osf.io/39nrj/.

#### 3.4.1 Method

##### 3.4.1.1 Participants

A power analysis using data from our previous experiments indicated that we needed to recruit around 350 participants to achieve 80% power to detect the hypothesized interaction.

Based on the inclusion rate of participants in the previous experiments, 448 participants were initially recruited on Amazon.com's Mechanical Turk and completed the experiment for $3 payment. We used the Turkolizer software from Gibson et al. (2011). The experimental tasks were available to participants who had (i) an IP address inside the United States, (ii) previously completed more than 500 tasks on Mechanical Turk, (iii) an approval rate above 98%, and (iv) had not participated in similar experiments before.

If a participant's accuracy on comprehension questions (including those in the target trials and those in the exposure trials) was less than 60%, we rejected their work and reposted it so that a new participant could receive it. (We explicitly stated at the beginning of each experimental task that “Because some Mechanical Turk users answer questions randomly, we will reject users with error rates of 25% or larger.” In practice, we rejected participants with error rates of 40% or larger.)

Design, materials, and procedure were similar to Experiment 2, except for the following changes in the materials and procedure.

##### 3.4.1.2 Materials

We revised the materials so that each pair of “no numerals” and “with numerals” exposure trial varied minimally in only two ways: (1) whether or not numerals were used when describing how many items were checked. (i.e., “I have now checked many of the tables” vs. “I have now checked 10 of the 12 tables.”), and (2) whether or not numerals were used in the conclusion the speaker made (i.e., “I can tell you that the accuracy of today's quizzes has been improved a lot” v. “I can tell you 2 of today's quizzes have errors in them.”). Specifically, we revised the exposure trials in Experiment 4 in the following three aspects:

- In Experiment 2, there were minor mismatches between “no numerals” and “with numerals” exposure trials regarding how many items in total the speaker needed to check. For example, when a “no numerals” exposure says “Kim needs to check whether 7 pieces of ceramics are correctly polished,” its “with numerals” counterpart should also say “Kim needs to check whether 7 pieces of ceramics are correctly polished,” instead of “6 pieces of ceramics.” There were six cases of this type of mismatch in the materials of Experiment 2. We revised them all in Experiment 4.
- In Experiment 2 and Experiment 3, there were minor mismatches between “no numerals” and “with numerals” exposure trials regarding how many items the speaker checked. For example, when a “no numerals” exposure says a speaker has checked “many of the” items, its “with numerals” counterpart should say a speaker has checked more than half of the items using numerals. For example, if a “no numerals” exposure says “Chris has checked many of the markers,” its “with numerals” counterpart should say “Chris has checked 8 of the 10 markers,” instead of “4 of the 10 markers.” There were five cases of this type of mismatch in the materials of Experiment 2 and Experiment 3. We revised them all in Experiment 4.
- In Experiment 2 and Experiment 3, there were minor mismatches between “no numerals” and “with numerals” exposure trials regarding the affect of the speaker's conclusion. For example, if in a “with numerals” exposure, Robert checked 6 of the 12 windows and said six of today's windows are closed, then in the corresponding “with numerals” exposure, Robert should say “the result is not surprising,” instead of “the result is very surprising.” There were four cases of this type of mismatch in the materials of Experiment 2 and nine cases in Experiment 3. We revised them all in Experiment 4.

In addition, we also resolved the following two issues in the materials of Experiment 4:

- In Experiment 2 and Experiment 3, there were possible violations of the opening assumptions in the materials. All trials in our experiments begin with an opening assumption “[Some items] almost always have [some property]” (e.g., “The artifacts in Carina's art museum are almost always correctly documented.”) However, this opening assumption might be violated, if there are many exposure trials in which not all observed items are stated to have the property by the speaker (e.g., If the speaker has checked five of the five artifacts and says four of the artifacts are correctly documented.) In Experiment 4, we reduced the number of those exposure trials (namely, the trials in which not all the observed items had the property) to 6 out of 24. (In Experiment 2, there were 12. In Experiment 3, there were 10.)
- In Experiment 2 and Experiment 3, Yes and No answers to the comprehension questions were not evenly distributed. In Experiment 4, we counterbalanced Yes and No answers across both questions for all the trials.

##### 3.4.1.3 Procedure

Regarding the recruitment procedure, we used a more lenient data exclusion threshold. As in previous experiments, we excluded any participants (i) whose native language was not English (based on self‐report) and (ii) who did not come from the United States (based on self‐report and IP address). In a post‐hoc analysis of Experiments 1–3, we found that lowering the accuracy requirement on comprehension questions from 100% on critical trials (16 of 16) to 87.5% across the experiment (42/48 comprehension questions correct for E1; 56/64 for E2 and E3) resulted in similar statistical inferences as with the more restrictive cutoff. In order to include a greater proportion of participants in the analysis, we, therefore, lowered the inclusion threshold to 87.5% for Experiment 4. Any critical trials for which participants did not answer the corresponding comprehension questions correctly were also excluded.

#### 3.4.2 Results

There were 390 participants left after the data exclusion procedure. Fig. 1(e) shows the mean rating of the probability of *not all* items having the property across knowledgeability conditions and in both exposure conditions.

Effects of speaker knowledgeability and exposure to numerals on probability ratings were assessed via linear mixed‐effects regression using the lme4 package in R (Bates et al., 2015). Knowledgeability (partial = –0.5, full = 0.5) and exposure (no numerals = –0.5, numerals = 0.5), along with their interaction, were entered as contrast‐coded fixed effects. Varying intercepts were included for participants and items with varying slopes for knowledgeability by participants and varying slopes for all effects of interest (the two main effects and their interaction) by items.^9^ Table 5 shows the regression table of the fixed effects (see Table A1 in the online Appendix for random effects).

**Table 5:** The regression table of the fixed effects of Experiment 4

	Estimate	Std. error	df	*t*‐value	*p*‐value
Intercept	54.920	1.424	58.097	38.559	<2e‐16
Knowledgeability	14.235	1.340	83.316	10.623	<2e‐16
Exposure (numerals)	1.442	2.479	288.618	0.582	.561
Knowledgeability × Exposure	–6.901	2.764	38.261	–2.497	.017

As in Experiments 1–3, the probability ratings were lower in the partial knowledge condition (*M* = 47.95, *SD* = 30.16) than in the full knowledge condition (*M* = 62.18, *SD* = 33.77; *β* = 14.24, *SE* = 1.34, *t* = 10.62, *p* < .001).

There was no main effect of the contextual numerals on the probability ratings (*β* = 1.44, *SE* = 2.48, *t* = 0.58, *p* = .56).

Crucially, the interaction between speaker knowledgeability and contextual numerals was significant (*β* = –6.90, *SE* = 2.76, *t* = –2.50, *p* = .017). There was a larger effect of knowledgeability on scalar implicature in the context without numerals than in the context with numerals.^10^

We also conducted follow‐up tests of the effect of contextual numerals within each knowledgeability condition by dummy‐coding the knowledgeability conditions.^11^ When the speaker had only partial knowledge, the probability ratings were marginally significantly higher in the context with numerals than in the context without numerals (*β* = 4.89, *SE* = 2.55, *t* = 1.92, *p* = .056). When the speaker had full knowledge, the probability ratings in the context with numerals were not significantly different from those in the context without numerals (*β* = –2.01, *SE* = 3.01, *t* = –0.67, *p* = .51).

#### 3.4.3 Discussion

The probability ratings were lower in the partial knowledge condition than in the full knowledge condition, thus replicating Experiments 1–3 and G&S, as predicted by our RSA model.

This experiment also provided evidence for the predicted interaction between knowledgeability and alternative availability. The follow‐up tests furthermore showed that the scalar implicature was equally strong in the full knowledgeability conditions, but stronger in the partial knowledge case when numeral alternatives were made salient. These results were all in line with the predictions of our RSA model.

## 4 General discussion

G&S observed context effects on scalar implicature that have important ramifications for theories of meaning and inference: the knowledge state of a speaker affects what producing the word “some” means to a comprehender; the comprehender is less likely to derive the “some but not all” implicature of “some,” when the speaker only has partial knowledge. Degen and Tanenhaus (2016) also noticed the role context played in the processing of scalar implicature: the alternative words that the speaker uses (e.g., number words) in the context affects the speed of making a scalar implicature of “some.” In light of these results, we have investigated the effects of both speaker knowledgeability and the speaker's use of alternative words for “some” (e.g., number words) on the comprehender's final interpretation of “some.”

We first showed that a natural extension of the model proposed by G&S predicts that having alternative words available increases the strength of scalar implicature in the partial knowledge condition. That is, if the speaker has only opened two of the three letters, and also often uses words like “two” or “three” in the surrounding context, then the listener should infer “not all” more than in the baseline partial knowledge context. To test the model's prediction, we conducted four preregistered experiments. Table 1 shows a summary of the model's predictions, the crucial variables in the experimental design, and the experimental results. In all experiments, we manipulated two types of context: speaker knowledgeability and speaker's alternative utterances. Experiment 2 differed from Experiment 1 in that it had eight more exposure trials. Experiment 3 differed from Experiment 2 in that the range of the numerals in the context was narrower (1–5 for Experiment 3 vs. 2–22 for Experiment 2). Experiment 4 was a replication of Experiment 2, with a different recruitment process and minor revision of the materials.

All four of the experiments reported here replicated G&S's context effect (i.e., the knowledgeability effect). Hence, one contribution of this paper is to provide more support for the context effect on the interpretation of “some,” with our materials and method showing stable replicability of the effect. Even more importantly, this project has extended the knowledgeability effects that G&S observed, showing that the words that people use affect the strength of scalar implicatures that comprehenders make. We show that there is an interaction between speaker knowledgeability and speaker's use of numeral alternatives in the context: when the speaker has partial knowledge about the contents of the materials that they wish to talk about, comprehenders think that “some” is more likely to mean “not all” when the speaker also produces relevant alternative numbers, like “one,” “two,” and “three.” This interaction is exactly what was observed in Experiments 1, 2, and 4 (the interaction was marginal in Experiment 1). The power analysis at the beginning of Experiment 4 suggests that Experiments 1 and 3 are underpowered. The main effect of numerals in Experiment 3 is difficult to account for and is possibly an artifact.

The results provide further evidence for a social cognition view of implicature and have implications for theories of which alternative utterances are considered in pragmatic reasoning. G&S demonstrated that implicature is sensitive to the context and the knowledge of the speaker. The current experiments show that implicatures are also sensitive to the language that the speaker typically uses. This finding is consistent with prior work by Degen and Tanenhaus (2015, 2016), who demonstrated an effect of contextual numerals on the naturalness rating and response time associated with “some.” The current study extends that work, showing that when speaker knowledgeability is involved, changes to the set of alternative utterances made salient by the context can affect the final interpretation of a sentence—inferences about the speaker's intended meaning. These results highlight an important virtue of the probabilistic RSA framework: it naturally allows for the flexible integration of multiple sources of evidence about the speaker's mental states in drawing pragmatic inferences.

In contrast, Horn (1972), Katzir (2007), and Fox and Katzir (2011) posit that pragmatic alternatives are a function of the utterance that the speaker used and the language's grammar. These alternatives are algorithmically generated through substitution into the speaker's utterance. Most relevant for the current experiments, these theories do not posit that the surrounding context can modify the salience of alternatives, or the degree to which they are considered in pragmatic reasoning. While Fox and Katzir (2011) allow for context to enter these calculations in the form of focus sensitivity, this is a restrictive mechanism, and cannot account for the results of our experiments. The current experiments, therefore, provide evidence that these theories must be extended further to allow for a greater degree of context sensitivity in how alternatives are determined.

The current results also raise interesting questions about the relationship between different types of contexts which affect the derivation of implicature. Contextual numerals and speaker knowledgeability are both aspects of context, although they are different in nature. While numerals are arguably linguistic or language‐internal elements, knowledgeability is extra‐linguistic. We have shown that these kinds of factors work together to affect implicature.

The present work leaves open the question of *how* the set of alternatives is adapted to the context. It may be the case that participants immediately expand the set of alternatives they consider after experiencing a trial in which the speaker uses a numeral. Alternatively, they may gradually update their representation of alternatives to weigh certain candidate words more heavily over the course of the entire study. Analyses of order effects in the current experiments were not conclusive regarding these two possibilities, in part due to large variability across participants in the sequence of exposure and test trials. Designing studies to accurately measure the time course of adaptation effects in this paradigm is a promising avenue for future work which may shed light on the underlying mechanisms.

### Open Research Badges

This article has earned Open Data, Open Materials, and pre‐registered badges. Data and materials are available at https://osf.io/39nrj and pre‐registered are available at https://osf.io/ytpr5, https://osf.io/ujc3m, https://osf.io/93wbh, https://osf.io/w6h5b.

## Supporting information

Supporting Information

Supporting Information

## Notes

1 The maximal random effects structure was used (lmer syntax: Answer.prob ∼ knowledgeability * exposure + (1 + knowledgeability | participant) + (1 + knowledgeability * exposure | item)), but it resulted in a warning of a singular fit. The function “allFit” in lme4 was used to try the model‐fitting with all available optimizers. The results showed that all working optimizers converged to values that were practically equivalent (i.e., within 1% of each other). Hence, we considered the singularity warning to be a false positive and reported the regression results with the optimizer “bobyqa.”

2 An additional exploratory analysis, suggested by a reviewer (reported for this and the subsequent experiments), examined the effect of trial order. The centered and standardized version of the trial order variable and its interactions with all other variables were added to the model (lmer syntax: Answer.prob ∼ knowledgeability * exposure * trial_order_std + (1 + knowledgeability | participant) + (1 + knowledgeability * exposure | item). The main effect of trial order was not significant (*t* = 1.32) nor were any interactions with trial order (*t*s < 1).

3 The maximal random effects structure was used (lmer syntax: Answer.prob ∼ knowledgeability * exposure + (1 + knowledgeability | participant) + (1 + knowledgeability * exposure | item)), but it resulted in a warning of a singular fit. The function “allFit” in lme4 was used to try the model‐fitting with all available optimizers. The results showed that all working optimizers converged to values that were practically equivalent (i.e., within 1% of each other). Hence, we considered the singularity warning to be a false positive and reported the regression results with the optimizer “bobyqa.”

4 An additional analysis examined the effect of trial order. The centered and standardized version of the trial order variable and its interactions with all other variables were added to the model (lmer syntax: Answer.prob ∼ knowledgeability * exposure * trial_order_std + (1 + knowledgeability | participant) + (1 + knowledgeability * exposure | item). The main effect of trial order was not significant (*t* = –1.65) nor was the interaction of knowledgeability and trial order (*t* = –0.63). The interaction of exposure with trial order was significant (*t* = 2.08, *p* < .05), suggesting that in later trials, the effect of exposure to numerals may have increased probability ratings more than in earlier trials (across knowledgeability conditions). Crucially, the three‐way interaction of knowledgeability, exposure, and trial order was not significant (*t* = 1.09).

5 The maximal random effects structure was used (lmer syntax: Answer.prob ∼ knowledgeability * exposure + (1 + knowledgeability | participant) + (1 + knowledgeability * exposure | item)). The optimizer “Nelder–Mead Simplex” from “Nlopt” was used as the model converged with it.

6 The maximal random effects structure was used (lmer syntax: Answer.prob ∼ knowledgeability * exposure + (1 + knowledgeability | participant) + (1 + knowledgeability * exposure | item)). The optimizer “nmkbw” was used as the model converged with it.

7 The maximal random effects structure was attempted (lmer syntax: Answer.prob ∼ knowledgeability * exposure + (1 + knowledgeability | participant) + (1 + knowledgeability * exposure | item)), but it resulted in a warning of a singular fit. The function “allFit” in lme4 was used to try the model‐fitting with all available optimizers. The results showed that all working optimizers converged to values that were practically equivalent (i.e., within 1% of each other). Hence, we considered the singularity warning to be a false positive and reported the regression results with the optimizer “bobyqa.”

8 An additional analysis examined the effect of trial order. The centered and standardized version of the trial order variable and its interactions with all other variables were added to the model ( lmer syntax: Answer.prob ∼ knowledgeability * exposure * trial_order_std + (1 + knowledgeability | participant) + (1 + knowledgeability * exposure | item). The main effect of trial order was not significant (*t* = 1.75). The interaction of knowledgeability and trial order was significant (*t* = –2.89, *p*<.005), suggesting that the knowledgeability effect was reduced over the course of the experiment. The interaction of exposure with trial order was also significant (*t* = 2.70, *p* < .01), suggesting that in later trials, the effect of exposure to numerals may have increased probability ratings more than in earlier trials (across knowledgeability conditions). Crucially, the three‐way interaction of knowledgeability, exposure, and trial order was not significant (*t* = –0.48).

9 The maximal random effects structure was attempted (lmer syntax: Answer.prob ∼ knowledgeability * exposure + (1 + knowledgeability | participant) + (1 + knowledgeability * exposure | item)), but it resulted in a warning of a singular fit.The function “allFit” in lme4 was used to try the model‐fitting with all available optimizers. The results showed that all six working optimizers converged to values that were practically equivalent (i.e., within 1% of each other). Hence, we considered the singularity warning to be a false positive and reported the regression results with the optimizer “bobyqa.”

10 An additional analysis examined the effect of trial order. The centered and standardized version of the trial order variable and its interactions with all other variables were added to the model (lmer syntax: Answer.prob ∼ knowledgeability * exposure * trial_order_std + (1 + knowledgeability | participant) + (1 + knowledgeability * exposure | item). The main effect of trial order was not significant (*t* = 0.27). Nor were any interactions with trial order (*t*s < 1).

11 The maximal random effects structure was attempted (lmer syntax: Answer.prob ∼ knowledgeability * exposure + (1 + knowledgeability | participant) + (1 + knowledgeability * exposure | item)), but it resulted in a warning of a singular fit. The function “allFit” in lme4 was used to try the model‐fitting with all available optimizers. The results showed that not all working optimizers converged to values that were practically equivalent (i.e., not within 1% of each other). Hence, a simplified model was used, with the interaction term removed from the by‐item varying slopes (lmer syntax: Answer.prob ∼ knowledgeability * exposure + (1 + knowledgeability | participant) + (1 + knowledgeability + exposure | item)), but it still resulted in a warning of a singular fit. The results of “allFit” showed that all working optimizers converged to values that were practically equivalent (i.e., within 1% of each other). Hence, we considered the singularity warning to be a false positive and reported the regression results with the optimizer “bobyqa.”
