# Supplementary figures and images for: Scalar Implicature is Sensitive to Contextual Alternatives
Source: Cogn Sci. 2023 Feb 5;47(2):e13238. doi: 10.1111/cogs.13238 (PMC10078556; doi:10.1111/cogs.13238)

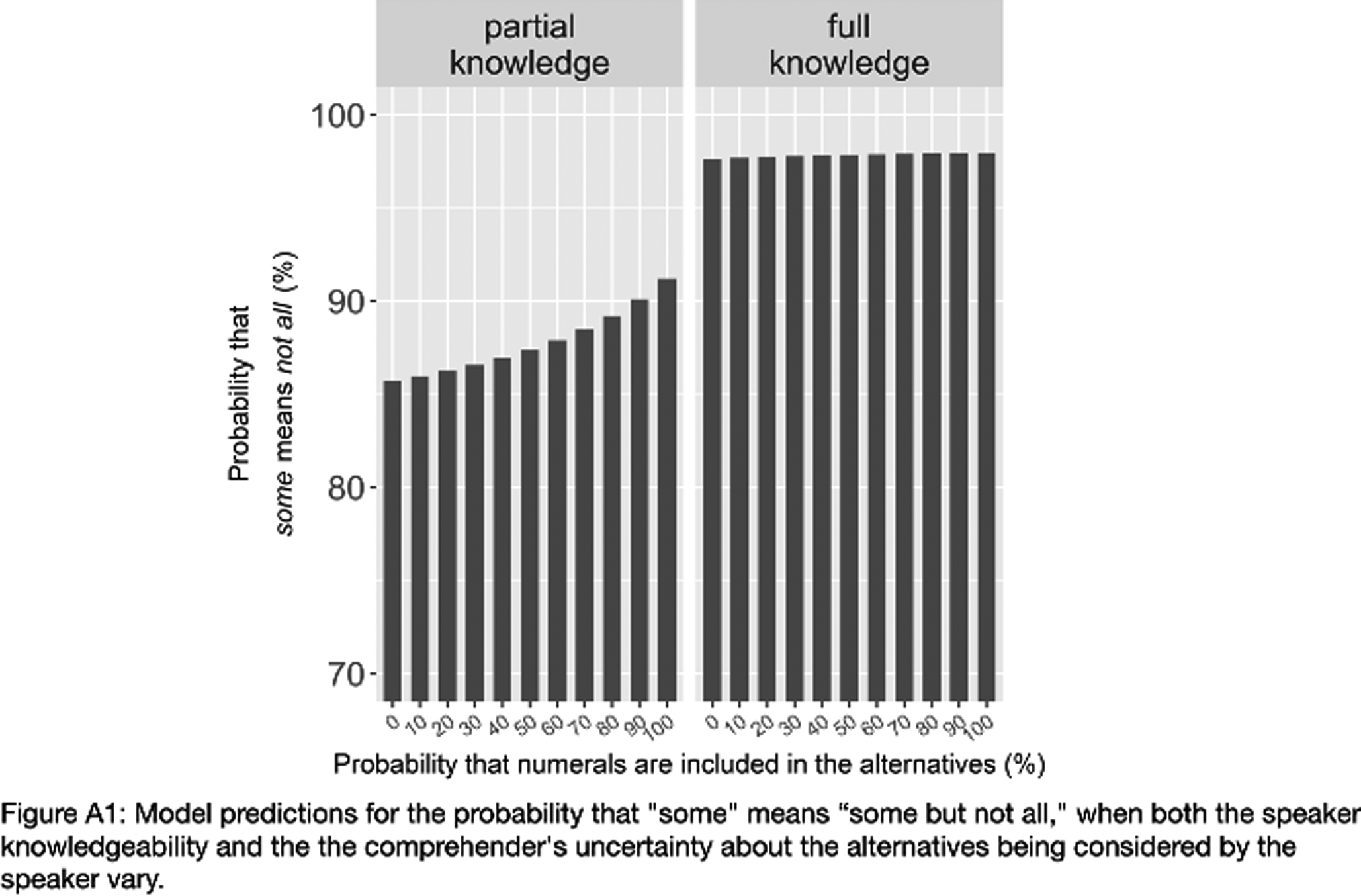

Supplement: Supplementary file 1 — Supporting Information [file COGS-47-0-s002.tiff]
