# Supplementary material for: Scalar Implicature is Sensitive to Contextual Alternatives
Source: Cogn Sci. 2023 Feb 5;47(2):e13238. doi: 10.1111/cogs.13238 (PMC10078556; doi:10.1111/cogs.13238)
Supplement: Supplementary file 2 — Supporting Information [file COGS-47-0-s001.docx]

| Random Effects | Std. Dev. | | | |
| --- | --- | --- | --- | --- |
|  | Exp. 1 | Exp. 2 | Exp. 3 | Exp. 4 |
| (Intercept) \| Participant | 22.664 | 24.087 | 24.125 | 22.999 |
| Knowledgeability \| Participant | 26.550 | 24.252 | 25.210 | 20.979 |
| (Intercept) \| Item | 1.989 | 1.391 | 2.798 | 2.085 |
| Knowledgeability \| Item | 0.878 | 1.717 | 1.470 | 1.124 |
| Exposure (numerals) \| Item | 2.816 | 2.431 | 0.322 | 1.270 |
| Knowledgeability x Exposure \| Item | 1.256 | 4.488 | 2.872 | 2.945 |
| Residual | 19.228 | 19.812 | 20.285 | 19.510 |

Table A1: The regression table of the random effects of Experiments 1, 2 3, and 4.
